# Mol* Viewer: modern web app for 3D visualization and analysis of large biomolecular structures

**DOI:** 10.1093/nar/gkab314

**Published:** 2021-05-06

**Authors:** David Sehnal, Sebastian Bittrich, Mandar Deshpande, Radka Svobodová, Karel Berka, Václav Bazgier, Sameer Velankar, Stephen K Burley, Jaroslav Koča, Alexander S Rose

**Affiliations:** CEITEC - Central European Institute of Technology, Masaryk University, Brno 625 00, Czech Republic; National Centre for Biomolecular Research, Faculty of Science, Masaryk University, Brno 602 00, Czech Republic; Protein Data Bank in Europe (PDBe), European Molecular Biology Laboratory, European Bioinformatics Institute (EMBL–EBI), Wellcome Genome Campus, Hinxton CB10 1SD, UK; Research Collaboratory for Structural Bioinformatics (RCSB), San Diego Supercomputer Center, University of California San Diego, San Diego, CA 92093-0743, USA; Protein Data Bank in Europe (PDBe), European Molecular Biology Laboratory, European Bioinformatics Institute (EMBL–EBI), Wellcome Genome Campus, Hinxton CB10 1SD, UK; CEITEC - Central European Institute of Technology, Masaryk University, Brno 625 00, Czech Republic; National Centre for Biomolecular Research, Faculty of Science, Masaryk University, Brno 602 00, Czech Republic; Department of Physical Chemistry, Faculty of Science, Palacký University Olomouc, Olomouc 771 46, Czech Republic; Department of Physical Chemistry, Faculty of Science, Palacký University Olomouc, Olomouc 771 46, Czech Republic; Protein Data Bank in Europe (PDBe), European Molecular Biology Laboratory, European Bioinformatics Institute (EMBL–EBI), Wellcome Genome Campus, Hinxton CB10 1SD, UK; Research Collaboratory for Structural Bioinformatics Protein Data Bank (RCSB PDB), Institute for Quantitative Biomedicine and Department of Chemistry and Chemical Biology, Rutgers, The State University of New Jersey, Piscataway, NJ 08854-8076, USA; Rutgers Cancer Institute of New Jersey, Rutgers, The State University of New Jersey, New Brunswick, NJ 08903-2681, USA; Research Collaboratory for Structural Bioinformatics Protein Data Bank (RCSB PDB), San Diego Supercomputer Center and Skaggs School of Pharmacy and Pharmaceutical Sciences, University of California San Diego, San Diego, CA 92093-0654, USA; CEITEC - Central European Institute of Technology, Masaryk University, Brno 625 00, Czech Republic; National Centre for Biomolecular Research, Faculty of Science, Masaryk University, Brno 602 00, Czech Republic; Research Collaboratory for Structural Bioinformatics (RCSB), San Diego Supercomputer Center, University of California San Diego, San Diego, CA 92093-0743, USA

## Abstract

Large biomolecular structures are being determined experimentally on a daily basis using established techniques such as crystallography and electron microscopy. In addition, emerging integrative or hybrid methods (I/HM) are producing structural models of huge macromolecular machines and assemblies, sometimes containing 100s of millions of non-hydrogen atoms. The performance requirements for visualization and analysis tools delivering these data are increasing rapidly. Significant progress in developing online, web-native three-dimensional (3D) visualization tools was previously accomplished with the introduction of the LiteMol suite and NGL Viewers. Thereafter, Mol* development was jointly initiated by PDBe and RCSB PDB to combine and build on the strengths of LiteMol (developed by PDBe) and NGL (developed by RCSB PDB). The web-native Mol* Viewer enables 3D visualization and streaming of macromolecular coordinate and experimental data, together with capabilities for displaying structure quality, functional, or biological context annotations. High-performance graphics and data management allows users to simultaneously visualise up to hundreds of (superimposed) protein structures, stream molecular dynamics simulation trajectories, render cell-level models, or display huge I/HM structures. It is the primary 3D structure viewer used by PDBe and RCSB PDB. It can be easily integrated into third-party services. Mol* Viewer is open source and freely available at https://molstar.org/.

## Graphical Abstract

**Graphical Abstract:**
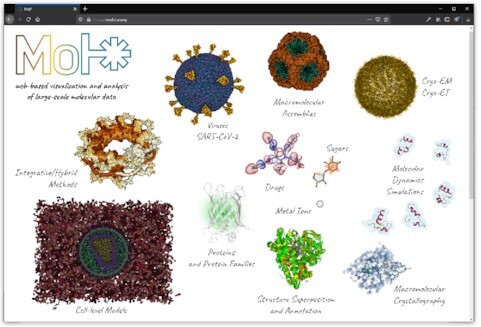
Overview of the large array of entities and systems that can be visualized and be manipulated with by the Mol* Viewer.

## INTRODUCTION

Experimental methods to determine the three-dimensional (3D) structures of biomolecules are continuously improved and produce molecular complexes from models with various resolutions that span multiple scales and can be dynamic throughout an experiment. By combining data from complementary experimental techniques including macromolecular crystallography (MX), nuclear magnetic resonance (NMR), 3D electron microscopy (3DEM), small-angle scattering (SAS), chemical crosslinking, or integrative/hybrid methods (I/HM), 3D structural models of large macromolecular systems can be determined (1,2). Such models include large macromolecular machines (3), dynamic assemblies, membrane organization (4), genome architecture (5,6) or even whole organelles (7). Access, visualization and analysis of these structures is a central part of structural biology and structural bioinformatics. However, as macromolecular data sets grow ever larger and more complex, it becomes challenging to create software tools to access, visualize, and manipulate them.

Web platforms, both mobile and desktop, have become an increasingly popular and essential tool for performing these tasks. Embracing advances in web browser technology provides the means for creating scalable molecular graphics and analysis tools with near-instant access to any available data. Web-based tools are platform-independent and require little or no local software installation, making them available to virtually everyone in both the scientific and non-scientific community, reaching an audience larger than ever before. Moreover, these technologies (most notably JavaScript, HTML and WebGL https://www.khronos.org/webgl/) and their surrounding ecosystem (including NPM https://www.npmjs.com/, Node.js https://nodejs.org/, TypeScript https://www.typescriptlang.org/, GitHub https://github.com/) offer good support for the development of modular libraries and components. In summary, the web provides a unique opportunity to develop a common library and a set of tools for accessing, analysing, and visualizing macromolecular data.

Here, we introduce Mol* (/’mol-star/) Viewer, part of the Mol* open source project (8) developed by an international group of contributors and supported by RCSB Protein Data Bank (RCSB PDB) (9) and Protein Data Bank in Europe (PDBe) (10) with the goal of developing a common library and tools for web-based molecular graphics and analyses. The Mol* project includes modules for data storage, in-memory representation, a query language, UI state management, visualization, and tools for efficient data access in a collaborative ecosystem. Collaborative development also helps with anticipating and keeping up with developments in structural biology and related fields as well as long-term sustainability.

The purpose of Mol* Viewer is to enable web-based molecular visualization and analyses by providing a common library for the rapid and efficient development of tools and services for the structural biology/bioinformatics community. Examples include showing experimental/validation-related data for macromolecular models; displaying various annotations for macromolecular models providing biological context, including SCOP (11), PFAM (12), UniProt (13); the creation of visually interesting, engaging, and interactive educational materials; or visualizing results from structural bioinformatics or computer-aided drug design efforts. The project builds on the code and knowledge the authors gained from developing web-based molecular viewers, analysis tools, and compressed file formats, including the LiteMol Suite (14), the NGL Viewer (15,16), PatternQuery (17), MMTF (18,19) and BinaryCIF (20). Mol* Viewer is developed as an open source project and hosted on GitHub (https://github.com/molstar).

## DESCRIPTION OF THE WEB APPLICATION

### Visualization capabilities

**Figure 1.**
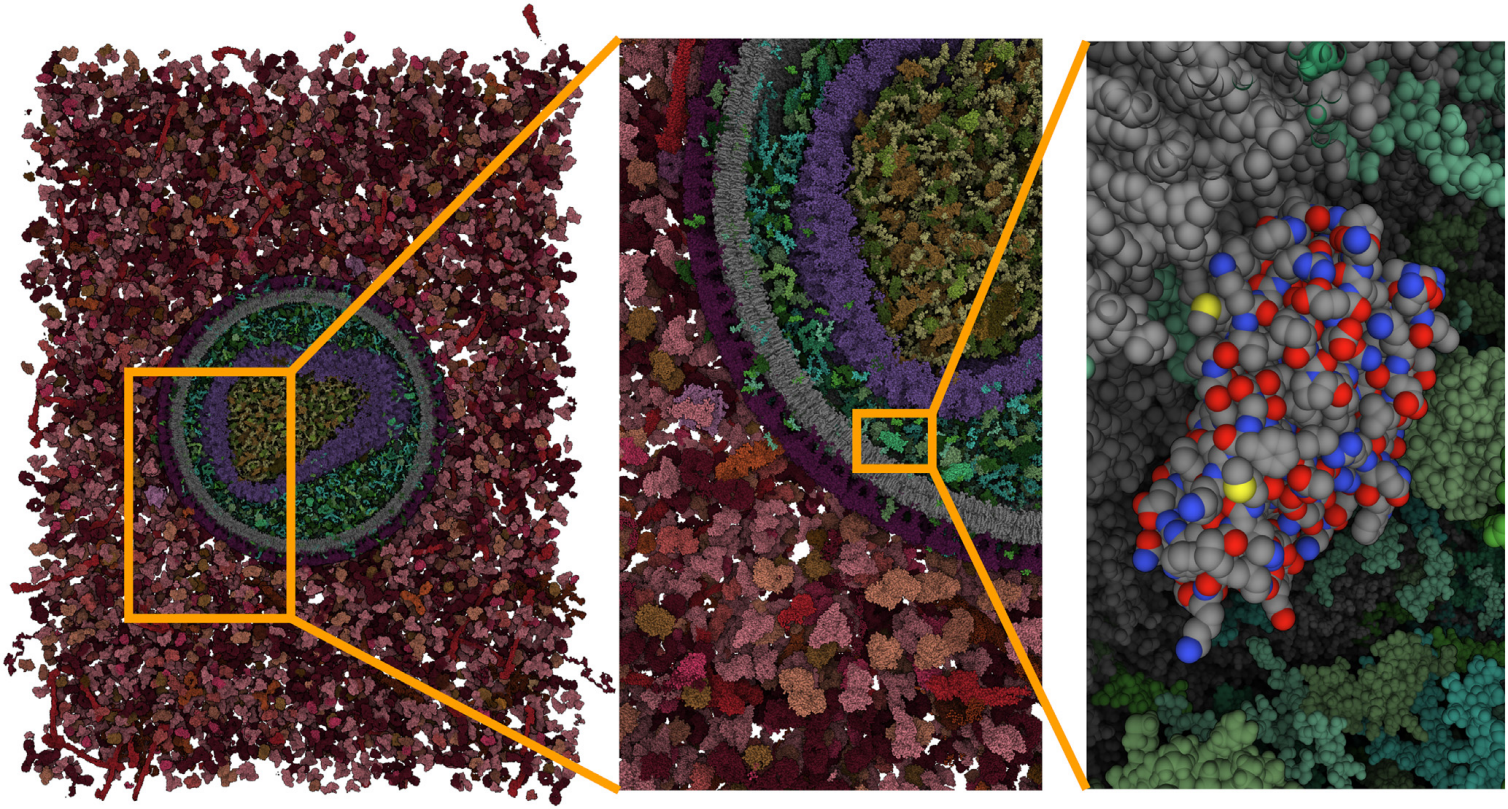
cellPACK model of enveloped HIV capsid in blood serum (67 870 140 atoms) with smooth browser-based visualization using nVidia RTX 2060 graphics card.

Mol* Viewer can visualize markedly larger molecular systems than other currently available web visualization tools. Due to built-in BinaryCIF (20), decompression support, and advanced techniques for model and volume/experimental data streaming (14), even large structures are interactively renderable over limited bandwidth. As such, Mol* Viewer is able to render many types of large systems, including ribosomes, virus capsids, collections of superimposed macromolecules (e.g. a comparison of individual members of the same protein family), or MD simulation systems. Additionally, it is able to visualize mesoscopic models such as cellPACK models, as illustrated in Figure 1.

### Overview of Mol* Viewer's new and improved features

**Figure 2.**
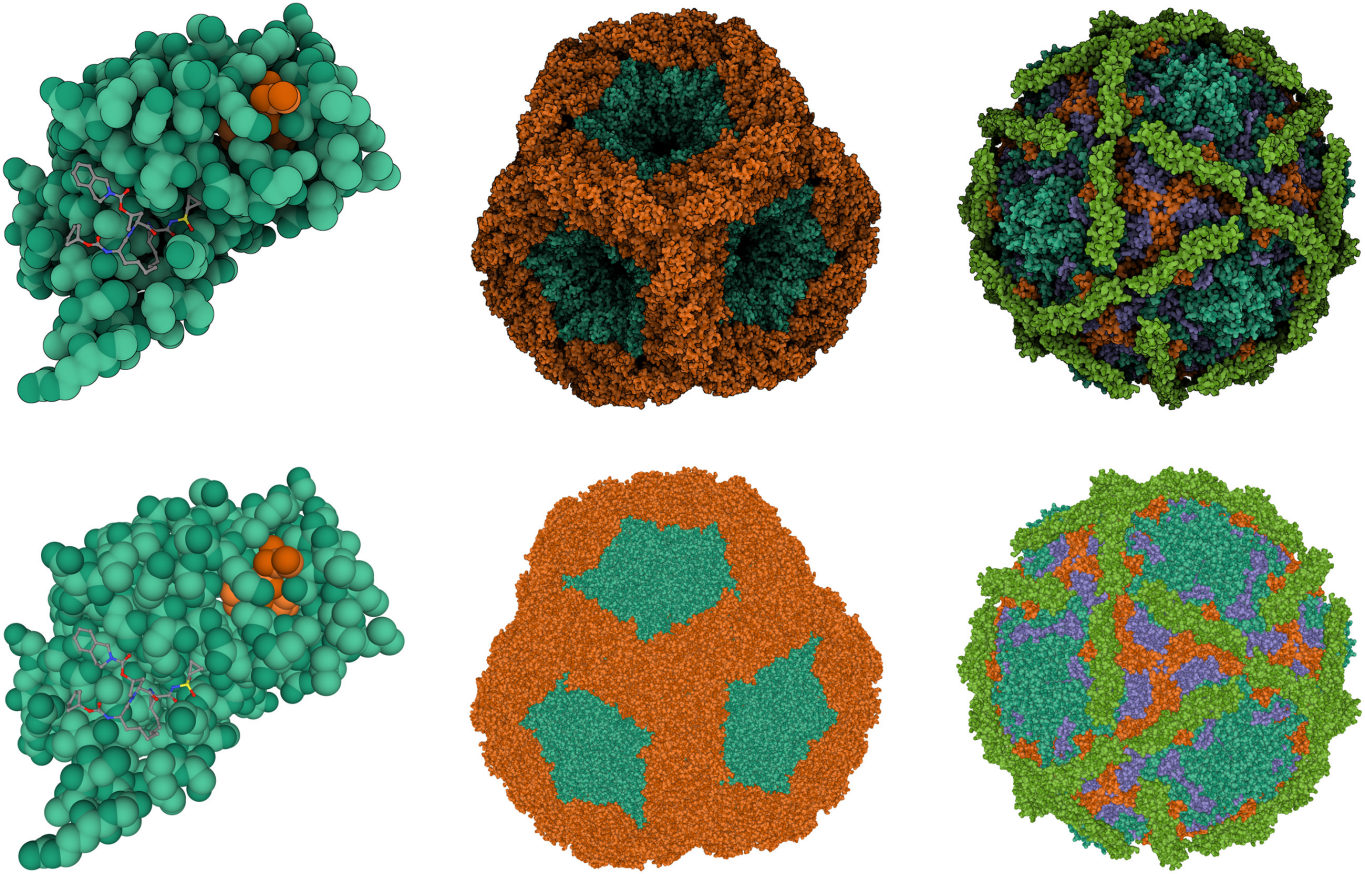
The effect of lighting modes and representations on the clarity of the visualization. PDB entries (left to right) 4ktc, 5fj5 and 1upn rendered using different lighting modes and representations. Top row: screen-space ambient occlusion and outline. Bottom row: direct light and ambient light. An interactive version of this figure is available at the Mol* Viewer web page (https://molstar.org/).

Mol* inherits many LiteMol suite and NGL Viewer features, as described in their respective articles. Here, we highlight some of its new and improved features:

- *Advanced User Interface:* access to many capabilities of the underlying Mol* library for fully creating molecular scenes with custom visuals and colourings.
- *High-quality rendering:* advanced rendering options for beautiful images and improved perception of details, such as lighting (matte, metallic, glossy, plastic), outlines, fog/depth cue, and ambient occlusion ‘shadows’ which darken crevices occluded from ambient light (Figure 2).
- *Sequence view and molecular component focus tools:* integrated sequence view and component (e.g. ligand or polymer) selection menu to help with navigating the structure and making selections.
- *Alignment of molecules:* sequence-guided pairwise alignment of two or more structures and ligand alignment by manual selection of corresponding atoms.
- *Measurements and labels:* geometric measurements (distances, angles, dihedrals) and their labelling.
- *Session state saving:* save the current molecular scene to reload it later.
- *Animation export:* video export of molecular dynamics simulations, rotating molecules, and 3D state transitions (such as zooming to a binding site).
- *Screenshots:* custom-sized, high-resolution, anti-aliased screenshots with preview, automatic cropping, and transparent background support.
- *Built-in data loading and annotations:* includes support for loading data from RCSB PDB and PDBe MX and 3DEM density servers, wwPDB validation reports, and RCSB PDB assembly symmetry.

### Data formats accepted by Mol* Viewer

Mol* Viewer is currently able to load and visualize many file formats with 3D structures:

- *Structures* (21): PDBx/mmCIF (https://mmcif.wwpdb.org/pdbx-mmcif-home-page.html) (including BinaryCIF encoded), pdb (ftp://ftp.wwpdb.org/pub/pdb/doc/format_descriptions/Format_v32_letter.pdf), pdbqt (http://autodock.scripps.edu/faqs-help/faq/what-is-the-format-of-a-pdbqt-file), gro (https://manual.gromacs.org/documentation/2018/user-guide/file-formats.html#gro), sdf (https://discover.3ds.com/sites/default/files/2020–08/biovia_ctfileformats_2020.pdf), mol (https://discover.3ds.com/sites/default/files/2020–08/biovia_ctfileformats_2020.pdf), mol2 (http://chemyang.ccnu.edu.cn/ccb/server/AIMMS/mol2.pdf), CIF-core (small molecule/crystallographic cif) (https://www.iucr.org/resources/cif/dictionaries/cif_core).
- *Volumes:* ccp4/mrc/map (22), dsn6/brix (http://svn.cgl.ucsf.edu/svn/chimera/trunk/libs/VolumeData/dsn6/brix-1.html), cube (http://paulbourke.net/dataformats/cube/), dx (https://www.ics.uci.edu/∼dock/manuals/apbs/html/user-guide/x2674.html), BinaryCIF (20).
- *Trajectories:* xtc (https://manual.gromacs.org/documentation/5.1/user-guide/file-formats.html#xtc), dcd (https://www.ks.uiuc.edu/Research/namd/2.14/ug.pdf), psf (topology) (http://www.ks.uiuc.edu/Training/Tutorials/namd/namd-tutorial-win.pdf).
- *Generic triangle geometries:* ply (e.g., coloured surfaces calculated by external tools) (http://gamma.cs.unc.edu/POWERPLANT/papers/ply.pdf).

Adding support for additional formats is usually a straightforward process.

### Implementation

TypeScript (https://www.typescriptlang.org/) was used for the development of the web application, WebGL (https://www.khronos.org/webgl/) for hardware-accelerated 3D rendering, and the standards of the open web platform (https://www.w3.org/standards/) for the whole tool. The React framework (https://reactjs.org/) was used to implement the application's UI.

## RESULTS

**Figure 3.**
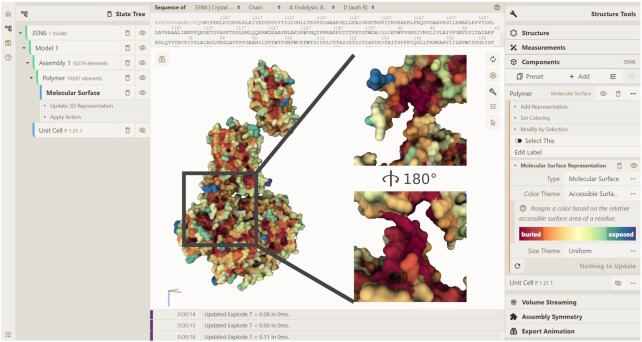
Mol* Viewer user interface. The 3D Canvas shows the beta2 adrenergic receptor-Gs protein complex (PDB entry 3sn6) with a molecular surface coloured by the relative solvent accessible surface area. The inset shows an exploded view of the deeply buried binding interface of the beta2 adrenergic receptor with the Gs-alpha subunit.

**Figure 4.**
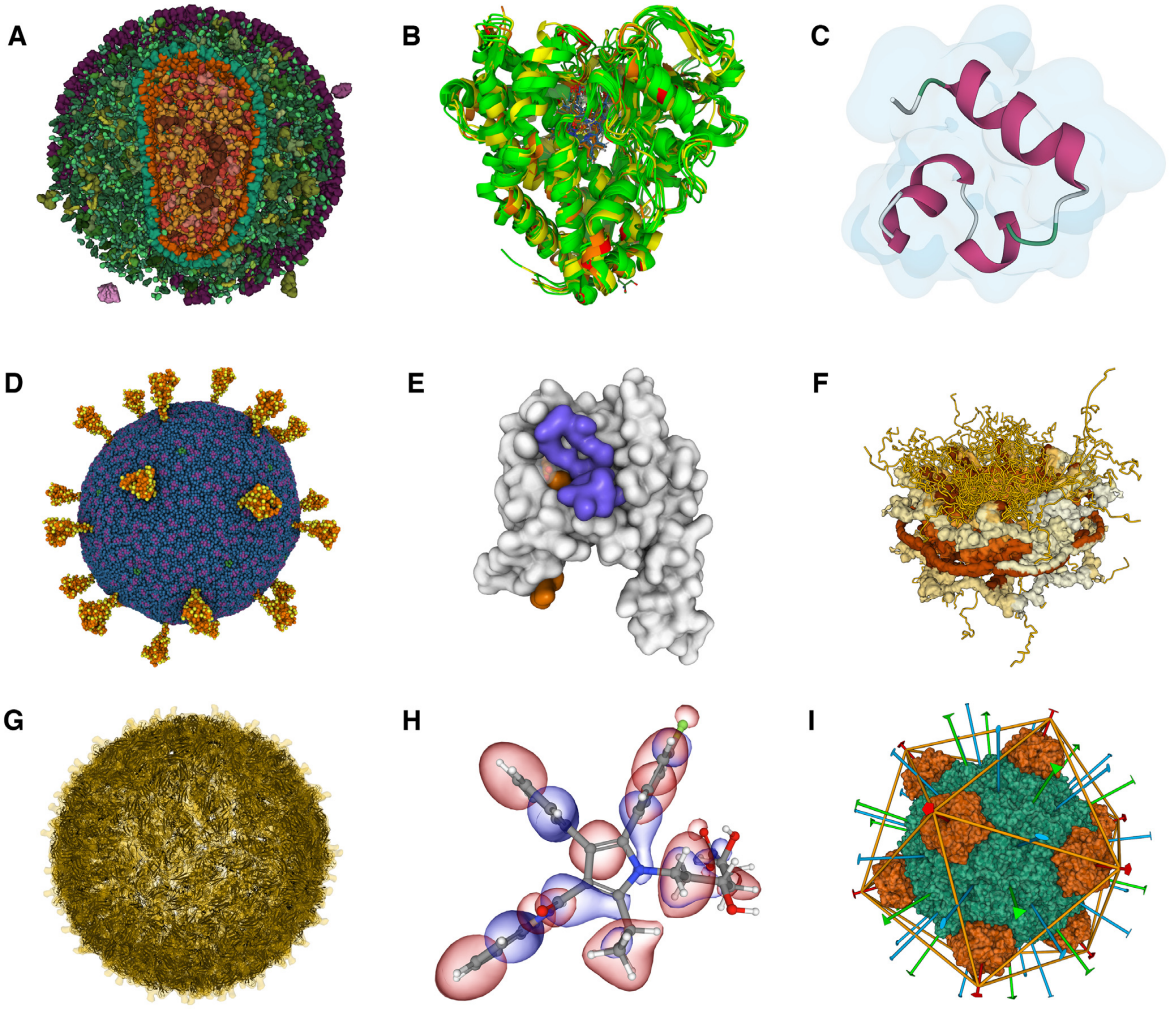
Practical demonstrations of Mol* Viewer visualization capabilities. We prepared several use cases that can be accessed from the Mol* web page (https://molstar.org). (**A**) Enveloped HIV capsid in blood serum. Originates from cellPACK and includes more than 13 million atoms. It is a simplified interactive version of Figure 1. (**B**) Superimposition of cytochromes P450. The superimposed structures are coloured according to a structure quality annotation based on wwPDB validation reports. (**C**) Villin un/folding simulation. A set of molecular dynamics trajectories for Markov state model analysis along villin headpiece folding and unfolding pathways. (**D**) SARS-CoV-2 Virion. The coarse-grained model composed of glycosylated S-proteins, M-proteins, E-proteins, and a lipid bilayer. (**E**) GAIN domain tethered agonist exposure simulation. Molecular dynamics trajectory. (**F**) Nuclear pore complex. Hybrid model originating from PDB-dev and including 238 288 unique residues. (**G**) Zika virus assembly including its Cryo-EM density. The original size of the data is about 1.6 GB. (**H**) Molecular orbitals and electron density of atorvastatin. Visualization of molecular orbitals and the electron density obtained using semiempirical GFN1-xTB method (34,35) (used at https://envision.entos.ai). (**I**) DNA binding protein assembly. A protein assembly is showcasing the RCSB PDB assembly symmetry annotation.

Mol* Viewer offers a wide variety of visualization aspects that are required by current structural biology needs. It can show one structure, a few structures, or a large set of structures (e.g. a whole protein family). The structures can be static or dynamic (e.g. a molecular dynamic trajectory). It can visualize large mesoscopic models (e.g. Genome3D (6), cellPACK models (23)), hybrid models (e.g. from PDB-Dev (1)), protein assemblies, but also residues at atomic resolution. All the levels of detail can be seamlessly navigated within one Mol* session (provided the availability of the data). Various visualization models of structure coordinates can be applied, specifically: surfaces and volumes (Gaussian surface, Gaussian volume, molecular surface, etc.), secondary structure (e.g. cartoon, ribbon), ligands (labels, glycan 3D-SNFG symbols (24), etc.), atoms (balls and sticks, lines, points, etc.). These visualization models can be coloured not only according to many types of atom properties (including occupancy, uncertainty, etc.), residue properties (including hydrophobicity and accessible surface area) and chain properties but also based on annotations (e.g. quality criteria annotations loaded from wwPDB validation reports (25)). Mol* also supports the rendering of electron densities and Cryo-EM maps. Further molecular characteristics, for example, molecular orbitals, non-covalent interactions, and membrane orientation, can be shown. Figure 3 shows the user interface of the Mol* Viewer and Figure 4 points to interactive practical demonstrations of Mol* Viewer's capabilities.

The high quality and applicability of Mol* Viewer was shown by a large number of integrations of it into scientific tools and databases. Mol* Viewer was integrated into PDBe and RCSB PDB as the primary 3D viewer where it is actively used by thousands of users daily. Moreover, Mol* Viewer was incorporated into many other resources, including PDBe-KB (26), PDB-Dev, EMDataResource (27), PED (28), MobiDB (29) and HARP (30).

## CONCLUSION

Mol* Viewer is a powerful web application for the visualization and analysis of molecular data. Its visualization capabilities far exceed other currently available web visualization tools. Mol* Viewer's speed and robustness allow the fast and intuitive visualization of molecular data ranging from atomistic models from PDB or MD simulations up to hybrid models with hundreds of thousands of residues, mesoscale cellPACK with tens of millions of atoms, or 3D Genome data. Furthermore, Mol* Viewer offers advanced selection and superimposition functionalities. It also offers a rich set of visualization models and colouring types. Last but not least, Mol* Viewer can save a visualization state operated by this web app.

Mol* Viewer can be used from its webpage https://molstar.org/ or can be integrated into other web applications. Its source codes are available on GitHub at https://github.com/molstar.

## DATA AVAILABILITY

Mol* Viewer is available for free at https://molstar.org/, under the MIT license, a permissive Open Source license, to facilitate code sharing and collaboration. The code is available on GitHub at https://github.com/molstar. Mol* Viewer can be readily embedded into any scientific web application.

Data for Figure 4C are available at https://doi.org/10.6084/m9.figshare.12040257.v1 and (31). Data for Figure 4D are available at (32). Data for Figure 4E are available at (33).

## References

[1] Burley S.K. , Kurisu G., Markley J.L., Nakamura H., Velankar S., Berman H.M., Sali A., Schwede T., Trewhella J. PDB-Dev: a prototype system for depositing integrative/hybrid structural models. Structure. 2017; 25:1317–1318. 28877501 10.1016/j.str.2017.08.001 PMC5821105

[2] Sali A. , Berman H.M., Schwede T., Trewhella J., Kleywegt G., Burley S.K., Markley J., Nakamura H., Adams P., Bonvin A.M.J.J. et al. Outcome of the first wwPDB hybrid/integrative methods task force workshop. Structure. 2015; 23:1156–1167. 26095030 10.1016/j.str.2015.05.013 PMC4933300

[3] Kummer E. , Ban N. Structural insights into mammalian mitochondrial translation elongation catalyzed by mtEFG1. EMBO J. 2020; 39:e104820. 32602580 10.15252/embj.2020104820 PMC7396830

[4] Chavent M. , Duncan A.L., Rassam P., Birkholz O., Hélie J., Reddy T., Beliaev D., Hambly B., Piehler J., Kleanthous C. et al. How nanoscale protein interactions determine the mesoscale dynamic organisation of bacterial outer membrane proteins. Nat. Commun. 2018; 9:2846. 30030429 10.1038/s41467-018-05255-9 PMC6054660

[5] Jung J. , Nishima W., Daniels M., Bascom G., Kobayashi C., Adedoyin A., Wall M., Lappala A., Phillips D., Fischer W. et al. Scaling molecular dynamics beyond 100,000 processor cores for large-scale biophysical simulations. J. Comput. Chem. 2019; 40:1919–1930. 30994934 10.1002/jcc.25840 PMC7153361

[6] Asbury T.M. , Mitman M., Tang J., Zheng W.J. Genome3D: a viewer-model framework for integrating and visualizing multi-scale epigenomic information within a three-dimensional genome. BMC Bioinformatics. 2010; 11:444. 20813045 10.1186/1471-2105-11-444 PMC2941692

[7] Singharoy A. , Maffeo C., Delgado-Magnero K.H., Swainsbury D.J.K., Sener M., Kleinekathöfer U., Vant J.W., Nguyen J., Hitchcock A., Isralewitz B. et al. Atoms to phenotypes: molecular design principles of cellular energy metabolism. Cell. 2019; 179:1098–1111. 31730852 10.1016/j.cell.2019.10.021 PMC7075482

[8] Sehnal D. , Rose A.S., Koča J., Burley S.K., Velankar S. Byška J. , Krone M., Sommer B. Mol*: Towards a common library and tools for web molecular graphics. Proceedings of the Workshop on Molecular Graphics and Visual Analysis of Molecular Data. 2018; 29–33.

[9] Berman H.M. , Westbrook J., Feng Z., Gilliland G., Bhat T.N., Weissig H., Shindyalov I.N., Bourne P.E. The Protein Data Bank. Nucleic Acids Res. 2000; 28:235–242. 10592235 10.1093/nar/28.1.235 PMC102472

[10] Mir S. , Alhroub Y., Anyango S., Armstrong D.R., Berrisford J.M., Clark A.R., Conroy M.J., Dana J.M., Deshpande M., Gupta D. et al. PDBe: towards reusable data delivery infrastructure at protein data bank in Europe. Nucleic Acids Res. 2018; 46:D486–D492. 29126160 10.1093/nar/gkx1070 PMC5753225

[11] Andreeva A. , Kulesha E., Gough J., Murzin A.G. The SCOP database in 2020: expanded classification of representative family and superfamily domains of known protein structures. Nucleic Acids Res. 2020; 48:D376–D382. 31724711 10.1093/nar/gkz1064 PMC7139981

[12] Mistry J. , Chuguransky S., Williams L., Qureshi M., Salazar G.A., Sonnhammer E.L.L., Tosatto S.C.E., Paladin L., Raj S., Richardson L.J. et al. Pfam: The protein families database in 2021. Nucleic Acids Res. 2021; 49:D412–D419. 33125078 10.1093/nar/gkaa913 PMC7779014

[13] The Uniprot Consortium UniProt: the universal protein knowledgebase in 2021. Nucleic Acids Res. 2021; 49:D480–D489. 33237286 10.1093/nar/gkaa1100 PMC7778908

[14] Sehnal D. , Deshpande M., Svobodová Vařeková R., Mir S., Berka K., Midlik A., Pravda L., Velankar S., Koča J. LiteMol suite: interactive web-based visualization of large-scale macromolecular structure data. Nat. Methods. 2017; 14:1121–1122. 29190272 10.1038/nmeth.4499

[15] Rose A.S. , Hildebrand P.W. NGL Viewer: a web application for molecular visualization. Nucleic Acids Res. 2015; 43:W576–W579. 25925569 10.1093/nar/gkv402 PMC4489237

[16] Rose A.S. , Bradley A.R., Valasatava Y., Duarte J.M., Prlić A., Rose P.W. Web-based molecular graphics for large complexes. Web3D ’16: Proceedings of the 21st International Conference on Web3D Technology. 2016; 185–186.

[17] Sehnal D. , Pravda L., Svobodová Vařeková R., Ionescu C.-M., Koča J. PatternQuery: web application for fast detection of biomacromolecular structural patterns in the entire Protein Data Bank. Nucleic Acids Res. 2015; 43:W383–W388. 26013810 10.1093/nar/gkv561 PMC4489247

[18] Bradley A.R. , Rose A.S., Pavelka A., Valasatava Y., Duarte J.M., Prlić A., Rose P.W. MMTF—an efficient file format for the transmission, visualization, and analysis of macromolecular structures. PLoS Comput. Biol. 2017; 13:e1005575. 28574982 10.1371/journal.pcbi.1005575 PMC5473584

[19] Valasatava Y. , Bradley A.R., Rose A.S., Duarte J.M., Prlić A., Rose P.W. Towards an efficient compression of 3D coordinates of macromolecular structures. PLoS One. 2017; 12:e0174846. 28362865 10.1371/journal.pone.0174846 PMC5376293

[20] Sehnal D. , Bittrich S., Velankar S., Koča J., Svobodová R., Burley S.K., Rose A.S. BinaryCIF and CIFTools—lightweight, efficient and extensible macromolecular data management. PLoS Comput. Biol. 2020; 16:e1008247. 33075050 10.1371/journal.pcbi.1008247 PMC7595629

[21] Westbrook J.D. , Fitzgerald P.M.D Gu J. , Bourne P.E. Chapter 10 The PDB format, mmCIF formats, and other data formats. Structural Bioinformatics. 2009; Hoboken, New Jersey Wiley-Blackwell 271–291.

[22] Cheng A. , Henderson R., Mastronarde D., Ludtke S.J., Schoenmakers R.H.M., Short J., Marabini R., Dallakyan S., Agard D., Winn M MRC2014: extensions to the MRC format header for electron cryo-microscopy and tomography. J. Struct. Biol. 2015; 192:146–150. 25882513 10.1016/j.jsb.2015.04.002 PMC4642651

[23] Johnson G.T. , Autin L., Al-Alusi M., Goodsell D.S., Sanner M.F., Olson A.J. cellPACK: a virtual mesoscope to model and visualize structural systems biology. Nat. Methods. 2015; 12:85–91. 25437435 10.1038/nmeth.3204 PMC4281296

[24] Sehnal D. , Grant O.C. Rapidly display glycan symbols in 3D structures: 3D-SNFG in LiteMol. J. Proteome Res. 2019; 18:770–774. 30179493 10.1021/acs.jproteome.8b00473

[25] wwPDB consortium Protein Data Bank: the single global archive for 3D macromolecular structure data. Nucleic Acids Res. 2019; 47:D520–D528. 30357364 10.1093/nar/gky949 PMC6324056

[26] PDBe-KB consortium PDBe-KB: a community-driven resource for structural and functional annotations. Nucleic Acids Res. 2020; 48:D344–D353. 31584092 10.1093/nar/gkz853 PMC6943075

[27] Lawson C.L. , Patwardhan A., Baker M.L., Hryc C., Garcia E.S., Hudson B.P., Lagerstedt I., Ludtke S.J., Pintilie G., Sala R. et al. EMDataBank unified data resource for 3DEM. Nucleic Acids Res. 2016; 44:D396–D403. 26578576 10.1093/nar/gkv1126 PMC4702818

[28] Lazar T. , Martínez-Pérez E., Quaglia F., Hatos A., Chemes L.B., Iserte J.A., Méndez N.A., Garrone N.A., Saldaño T.E., Marchetti J. et al. PED in 2021: a major update of the protein ensemble database for intrinsically disordered proteins. Nucleic Acids Res. 2021; 49:D404–D411. 33305318 10.1093/nar/gkaa1021 PMC7778965

[29] Piovesan D. , Necci M., Escobedo N., Monzon A.M., Hatos A., Mičetić I., Quaglia F., Paladin L., Ramasamy P., Dosztányi Z. et al. MobiDB: intrinsically disordered proteins in 2021. Nucleic Acids Res. 2021; 49:D361–D367. 33237329 10.1093/nar/gkaa1058 PMC7779018

[30] Vedithi S.C. , Malhotra S., Skwark M.J., Munir A., Acebrón-García-De-Eulate M., Waman V.P., Alsulami A., Ascher D.B., Blundell T.L. HARP: a database of structural impacts of systematic missense mutations in drug targets of *Mycobacterium leprae*. Comput. Struct. Biotechnol. J. 2020; 18:3692–3704. 33304465 10.1016/j.csbj.2020.11.013 PMC7711215

[31] Doerr S. , Harvey M.J., Noé F., De Fabritiis G. HTMD: high-throughput molecular dynamics for molecular discovery. J. Chem. Theory Comput. 2016; 12:1845–1852. 26949976 10.1021/acs.jctc.6b00049

[32] Yu A. , Pak A.J., He P., Monje-Galvan V., Casalino L., Gaieb Z., Dommer A.C., Amaro R.E., Voth G.A. A multiscale coarse-grained model of the SARS-CoV-2 virion. Biophys. J. 2021; 120:1097–1104. 33253634 10.1016/j.bpj.2020.10.048 PMC7695975

[33] Beliu G. , Altrichter S., Guixà-González R., Hemberger M., Brauer I., Dahse A.-K., Scholz N., Wieduwild R., Kuhlemann A., Batebi H. et al. Tethered agonist exposure in intact adhesion/class B2 GPCRs through intrinsic structural flexibility of the GAIN domain. Mol. Cell. 2021; 81:905–921. 33497605 10.1016/j.molcel.2020.12.042

[34] Grimme S. , Bannwarth C., Shushkov P. A robust and accurate tight-binding quantum chemical method for structures, vibrational frequencies, and noncovalent interactions of large molecular systems parametrized for all spd-block elements (Z = 1-86). J. Chem. Theory Comput. 2017; 13:1989–2009. 28418654 10.1021/acs.jctc.7b00118

[35] Manby F.R. , Miller T.F. III, Bygrave P.J., Ding F., Dresselhaus T., Batista-Romero F.A., Buccheri A., Bungey C., Lee S.J.R., Meli R. et al. entos: a quantum molecular simulation package. 2019; ChemRxiv doi:26 February 2019, preprint: not peer reviewed 10.26434/chemrxiv.7762646.v2.

